# Methodological Components for Evaluating Intervention Effectiveness of SOS Feeding Approach: A Feasibility Study

**DOI:** 10.3390/children12030373

**Published:** 2025-03-17

**Authors:** Sarah A. Schoen, Rachel Balderrama, Emma Dopheide, Ariel Harris, Laura Hoffman, Samantha Sasse

**Affiliations:** 1 Research Department, STAR Institute for Sensory Processing, 6911 S. Yosemite Street, Centennial, CO 80112, USA; 2 Feeding Program, STAR Institute for Sensory Processing, 6911 S. Yosemite Street, Centennial, CO 80112, USA; rachel.balderrama@sensoryhealth.org; 3 Occupational Therapy Department, University of Colorado Health, Aurora, CO 80045, USA; emma.dopheide@gmail.com; 4 Developmental FX, Denver, CO 80205, USA; arielzharris@gmail.com; 5 CI Pediatric Centers, Middleton, WI 53562, USA; laurahoff456@gmail.com; 6 Desert Valley Pediatric Therapy, Phoenix, AZ 85044, USA; ssasse@dvpediatrictherapy.com

**Keywords:** pediatric feeding disorder, observational analysis, treatment outcomes, behavioral coding

## Abstract

**Background/Objectives**: There is a paucity of research that explores the effectiveness of the Sequential Oral Sensory (SOS) Approach to Feeding. The purpose of this feasibility study was to evaluate the necessary components for the implementation of a treatment effectiveness study on the Sequential Oral Sensory (SOS) Approach to Feeding. The primary aims were to develop a fidelity measure, determine the feasibility of video coding, create an observational coding scheme, and determine if the outcome measures were sensitive to change. **Methods**: Over a 4-year period, data were collected from twelve participants aged 4 to 8 years with developmental disorders, with the assistance of four occupational therapy doctoral students. A fidelity measure was created, and inter-rater reliability was established among the four coders. Videotapes were collected at home and in the clinic. A behavioral coding system, consistent with the SOS Steps to Eating hierarchy, was developed for scoring feeding behaviors. **Results**: The preliminary inter-rater reliability was reported, and the coding results were represented graphically. Two additional outcome measures were piloted—a visual analog scale (VAS) and the Parenting Stress Index (PSI). The VAS was sensitive to changes in each parent’s ability to support their child, as well as in each client’s progress. The PSI also showed sensitivity to changes in the decline of parent-reported stress and child stress indices. **Conclusions**: Findings demonstrate fidelity to the SOS Approach, as well as sensitive outcomes, using behavioral coding and parent-reported measures. These evidence-based tools and procedures offer researchers and clinicians objective and meaningful feeding outcomes.

## 1. Introduction

Eating is one of the most complex activities an individual can participate in, as multiple body systems must be engaged (e.g., sensory, motor, oral, interoceptive, psychological, emotional, and social). There has been growing awareness of feeding challenges affecting children with developmental and neurodevelopmental conditions [1]. The incidence of pediatric feeding disorders continues to rise and is one of the most common childhood challenges [2]. In fact, research has shown that some form of feeding disorder affects 30–45% of typically developing children [3] and up to 80% of children with developmental disabilities [4]. Prevalence estimates in children with autism range from 46% to 89% [5] and are also extremely common in children with sensory processing difficulties [6,7].

The importance of feeding challenges in pediatrics is further reflected by the inclusion of a new diagnostic term, *Pediatric Feeding Disorder* (PFD), introduced by The World Health Organization in 2020 and officially added to the ICD-10 in 2021 [8]. Feeding challenges not only impact a child’s daily functioning, development, and psychosocial function but also equally affect the family by negatively impacting the caregiver–child relationship [9], parental sense of competence [10], and overall caregiver stress [3,11]. These findings highlight the need for empirical research to support effective feeding interventions.

### 1.1. Connection Between Sensory Processing and Feeding

Children who have difficulty processing sensory information may be at risk of developing disordered feeding or feeding problems [6], since feeding is inherently a multisensory experience. While eating, individuals are perceiving and processing information from all sensory systems, including the gustatory, tactile, olfactory, and visual systems [6]. In fact, the current literature suggests that sensory processing difficulties and feeding problems can frequently co-occur in some children [5,6,12,13,14,15,16]. Studies have shown that children with atypical sensory processing experience more problems eating [5], including food refusal [12,13,14] and food selectivity [13,14]. Sensory qualities such as taste, texture, temperature, or color of foods may be reasons for food selectivity or food rejection [14].

Specifically, both sensory over-responsivity and sensory under-responsivity have been linked with a decreased variety of food intake and social enjoyment of eating [5]. Oral textural sensitivity was found to be a predictor of selective eating in both children and college students [16]. Additionally, sensitivity to taste and smell was a predictor of picky eating in typically developing children [15,17], as well as in children with Attention Deficit Hyperactivity Disorder, Autism Spectrum Disorder, and Tourette syndrome [15]. Sensory processing difficulties have also been linked to behavioral problems, including challenging feeder–child interactions and the refusal to engage in eating [6].

### 1.2. The SOS Approach to Feeding

The Sequential Oral Sensory (SOS) Approach to Feeding is an internationally recognized, multidisciplinary intervention designed to support the development of feeding skills in children and teens that are problem feeders or picky eaters (https://sosapproachtofeeding.com). The underlying principles of the SOS approach include using normal development as a blueprint for creating a therapy plan, as well as systematic desensitization when adverse reactions are present. One underlying theoretical premise of the SOS program is systematic desensitization, as well as addressing playful interactions and the exposure to new, varied foods and food textures depending on the sensory presentation. During feeding therapy sessions utilizing this approach, foods are introduced based on the developmental characteristics of the food type (e.g., purees are for developmentally younger children than hard, solid food). This is referred to as a ‘food hierarchy’, which is designed to support the development of oral motor skills, tolerance to the sensory properties of foods, and the expansion of one’s food repertoire. As a measurable progression of feeding abilities, the food hierarchy also lends itself naturally to behavioral coding as a treatment outcome measure, reflecting the progression in the successful acquisition of abilities and skills for eating [18].

Although limited, there is emerging evidence of the effectiveness of the SOS Approach to Feeding. A small, randomized, controlled trial demonstrated improved child feeding behaviors and parental behaviors in the intervention group following 28 sessions using the SOS approach [19]. Another promising study from Hsin et al., 2023, combined the SOS approach with two other interventions for families participating in an interdisciplinary group feeding program [20]. Although the outcomes supported fewer feeding problems and decreased parental strain, it is unclear as to which components of the SOS approach contributed to this positive change. A retrospective study of 34 participants (Benson et al., 2013) also provided preliminary data indicating that the SOS feeding program was beneficial for children with neurological impairments [18]. Peterson et al. (2016) explored a modified version of the SOS approach, showing a small effect and promising findings related to the generalization of eating behaviors to the home environment [21]. Additionally, the SOS approach has also been combined with other interventions such as behavior modification and has been found to improve the food acceptance of previously refused foods [22]. Lastly, in a case study utilizing the SOS approach, a child increased the variety of foods ingested, improved their oral motor skills, and had a greater ability to eat independently [23]. These findings demonstrate early evidence and the need for more research.

### 1.3. Feasibility and Pilot Research

In preparation for conducting a randomized controlled trial (RCT) of treatment effectiveness, the current study focused on feasibility questions, and the pilot research was designed to accelerate the process of application to an RCT [24]. The feasibility aspects of this study addressed methodology questions and the data collection of potential outcomes. The objectives were to assess the sample characteristics, refine the data collection procedures, and identify sensitive outcome measures. Additionally, this study sought to examine potential probes for a graphic representation of change across intervention. The specific goals were to report on features such as the inclusion and exclusion criteria for participants and the design rationale, as well as to operationalize the definitions of the target behaviors [25].

Thus, the purpose was to develop, employ, and recommend procedures for a rigorous study of effectiveness of the SOS Approach to Feeding and share the lessons learned. The aims were as follows:

1. Develop a fidelity measure for the SOS Approach to Feeding;
2. Identify objective probes/behaviors that could be used in a future treatment effectiveness study;
3. Identify sensitive outcome measures from participation in an intensive feeding program.

## 2. Method

This feasibility/pilot study evaluated the necessary components for the implementation of a treatment effectiveness study. Each component from the specific aims is described below. The Institutional Review Board at Rocky Mountain University of Health Professions approved this study.

### 2.1. Participants

Participants were recruited for each of the different aims of this study. The inclusion criteria for this pilot study were children between the ages of two and eleven years who were scheduled to participate in an intensive feeding at a private clinic in western United States. De-identified treatment session videos were used for the development and reliability testing of the fidelity measure, as well as for the development and reliability of the behavioral coding scheme. There was a total of 12 participants. Four participants were recruited to determine the feasibility of collecting home videos and for the identification of additional probes. Home videos were submitted for the identification of behaviors interfering with daily meals. Two additional participants were then recruited to determine the feasibility of collecting clinic videos and for calculating the inter-rater reliability of the behavioral coding scheme. Lastly, six participants were recruited for the collection of preliminary data on sensitivity of measures pre- and post-intervention. These participants were drawn from the existing caseload of feeding therapists at the clinic. See Table 1.

### 2.2. Investigators/The Research Team

The research team consisted of two principal investigators and four occupational therapy doctoral students who served as research assistants for the inter-rater reliability components of the study. One of the PIs was the Director of the Research Department and the other was the Feeding Program Coordinator. The four students were in the last year of their clinical doctorate programs in occupational therapy. Each spent 14 weeks completing their Capstone project in support of this study.

### 2.3. Description of the Intervention

The intervention employed in this study is the SOS Approach to Feeding for children with co-existing feeding and developmental sensory integration needs. This program uniquely supports children’s nutritional and social growth and development, while providing parents with knowledge, tools, and strategies for carryover at home. During feeding therapy sessions utilizing the SOS Approach to Feeding, a food hierarchy is used to support the development of oral motor skills, tolerance of the sensory properties of foods, and expansion of one’s food repertoire.

### 2.4. Development of Fidelity Measures

A fidelity measure was created for the SOS Feeding Approach to be consistent with the key features of the SOS Approach to Feeding [26] as employed at the private clinic in which the therapy was delivered. The purpose was to provide a reliable measure that monitored compliance and consistency with the approach.

Videotaped treatment sessions were used to score the fidelity measure and establish inter-rater reliability. After each student independently viewed the videos, their scores were compared to those of the principal investigators, and any areas of disagreement were resolved. Data analysis was conducted using Fleiss Multi-Rater Kappa to determine inter-rater reliability.

### 2.5. Identification of Outcome Behaviors and Feasibility of Video Coding

Procedures were piloted in order to determine the feasibility of video coding in the clinic and during home mealtimes, as well as to establish potential outcome behaviors. Clinic behaviors were assessed using the Steps to Eating Food Hierarchy of the SOS approach [27]. This hierarchy encompassed 32 steps that have been described as the components of independent eating [27]. The home meals were evaluated based on the parental priorities for intervention. The parental priorities were obtained during the parent intake interviews conducted prior to initiating intervention. For the home videos, families were asked to send the researchers three to five 10 min videos of their child during a typical mealtime. Due to the individual nature of the home mealtime videos, additional probes were generated by clinicians based on the review of the baseline home videos. A collaborative goal-setting session allowed for the therapist’s observations of each child’s feeding challenges to be combined with their parents’ priorities for change to generate and operationalize measurable treatment outcomes, resulting in two to three goals. A 10 min video of a typical home meal was collected once a week while each child was receiving treatment.

The clinic videotaping involved data collection before the initiation of intervention and then weekly during the intervention phase. Based on a review of the information provided by each family, five novel foods were selected and presented at each baseline and intervention session. Each child’s feeding-related behaviors were scored using the food hierarchy coding scheme described below. This procedure was repeated twice a week during the intervention phase, on a day prior to that week’s intervention session.

#### Development of the Behavioral Coding Scheme

A behavioral coding scheme was developed based on the Steps to Eating Food Hierarchy. There were 32 coded steps, which were organized into six broad categories that classify the progression of feeding skills, such as tolerating, interacting, smelling, touching, tasting, and eating foods [27]. These categories were used to describe the level of engagement of the child with respect to the foods presented. The specific codes rated engagement with the food based on a scale ranging from the lowest level (tolerates food in room) to the highest level (bites food, chews it, swallows all, or takes puree liquid into mouth and swallows).

A similar coding method has been used to evaluate the SOS Approach to Feeding in a previous retrospective study [18]. A complete list of the behavioral codes and their description is available upon request. 

Videos were coded in 10 s increments. If the participant engaged with one of the foods during a 10 s increment, the matching category of the interaction was coded as a “1”. The coding convention of “1” plus (+) the letter abbreviation of the corresponding food was used to denote which food the interaction reflected. If the participant interacted with all the foods at once for a certain category, this was coded as “1all”.

Videos were randomly assigned a four-digit number, so that coders would be blind to the order of the sessions. Four videos were chosen at random and coded individually by two of the four student assistants trained in the coding system. The length of the videos varied; in order to provide consistency, the number of coded interactions was divided by the total number of possible identified interactions for each video.

Inter-rater reliability was computed for the four videos by calculating the percentage agreement using the following formula: agreement/(agreement + disagreement) × 100.

### 2.6. Sensitivity of Outcome Measures

#### Pretest Posttest Measures

Specific pre-test and post-test measures were selected for this study to capture each parent’s perspective on changes in their child’s feeding-related behavior, as well as the parent-reported changes in knowledge, experience of stress, and sense of competence. Measures that were piloted to capture this information included the Parenting Stress Index [28] and a visual analog scale (VAS) that contained questions based on clinical experience and informal feedback from families after finishing a feeding therapy program.

The Parenting Stress Index, Fourth Edition (PSI™-4, [28]), is a parent-report questionnaire that has been used in research to address the psychosocial concerns of a family [29]. The PSI comprises 101 questions that demonstrate the amount of stress in the parent–child system. The child domain evaluates the sources of stress as gathered from the parents’ reports of the children’s characteristics, while the parent domain measures the sources of stress related to the parents’ characteristics. Both domains were piloted in this study—the child domain which contained six subscales (e.g., distractibility/hyperactivity (DI), adaptability (AD), reinforces parent (RE), demandingness (DE), mood (MO), and acceptability (AC)) and the parent domain, which consisted of seven subscales (e.g., competence (CO), isolation (IS), attachment (AT), health (HE), role restriction (RO), depression (DP), and spouse/parenting partner relationship (SP)). Lower scores reflected less stress. Good reliability and validity have been reported. Internal consistency ranged from 0.78 to 0.88 for the child domain subscales and 0.75–0.87 for the parent domain subscales. Strong validity has also been reported across the subscales [28]. Due to the preliminary nature of this study, the subscales were the primary focus.

The visual analog scale (VAS) employed in this study consisted of 12 questions. Given that parental participation is central to the SOS approach, the VAS questions were worded to reflect the child outcomes and the parent outcomes. Both outcomes were considered essential to measuring the treatment effectiveness of this intervention. Thus, the VAS was used to determine the children’s progress during therapy in addition to the parental gains in knowledge, competency, and their satisfaction with the intervention. Specifically, the questions ranged from the child’s ability to learn about new foods through exploration or play to the parents’ understanding of their child’s strengths and challenges, as well as their perceived ability to implement tools and strategies to support their child’s feeding skill development or feeding-related behaviors.

A VAS is a 100 mm long horizontal line used to measure subjective variables such as behaviors or attitudes [30]. Descriptor words representing opposite extremes are at either end of the line; a mark is placed between the words by the respondent, demonstrating the participants’ perceived status [31]. The scale ranged from “none of the time” to “all of the time”. Higher scores reflected the positive end of the line, indicating more time displaying the behavior. The VAS has been found to be a reliable and valid measure [31]. Recently, the VAS has also been utilized as an outcome measure and deemed feasible for documenting treatment effectiveness [32].

### 2.7. Preliminary/Pilot Data

Following the development and establishment of reliability of the fidelity measure and the behavioral coding scheme, preliminary data were collected on these measures to establish feasibility of visually representing the data to reflect change. The data were not designed to reflect treatment effectiveness, as the baseline data could not be collected. Data from the video coding were analyzed/presented graphically. Additionally, pre- and post-intervention data were collected using the PSI and the VAS. A paired samples Wilcoxon signed-rank test was used to compare the pre-test and post-test measures.

## 3. Results

### 3.1. Fidelity (Inter-Rater Reliability)

A fidelity measure was created based on the principles of the SOS Approach to Feeding, as articulated by Dr. Toomey and acquired through continuing education trainings. The measure was found to have a moderate to very good inter-rater reliability. The kappa value for the fidelity measure increased in strength of agreement from 0.56 to 0.79 with the clarification and refinement of the operational definitions following the data collection.

### 3.2. Identification of Probes and Feasibility of Videotaping

Home videos were submitted by four families and reviewed by the research team. Due to the individual nature of the home mealtime videos, additional probes were generated based on the review of the home videos; observations of the child’s feeding challenges were combined with the parents’ priorities for change in order to generate measurable treatment outcomes. Table 2 presents the suggested probes/behaviors for four clients based on their reported and observed challenges. Notably, the families had difficulty submitting the requested number of videos both prior to and during intervention.

Clinic probe outcomes were based on a review of the intake information collected from each family. Based on this information, the primary clinician successfully identified five novel foods and the appropriate treatment outcomes consistent with the Stages of Eating Food Hierarchy. The videotaping was intended to be unobtrusive so that the feeding-related behaviors could be scored from the videos. An attempt was made to collect clinic video data on two children. However, participant 6 did not tolerate the clinic videotaping after the first two sessions, so his data could not be compiled or reported. From a procedural feasibility standpoint, only three clinic probes were collected prior to the initiation of intervention for participant 5 due to the urgency to start intervention. Thus, findings cannot be reported within the context of a single-subject study. This child also became resistant to being observed during the intervention phase of data collection. Additionally, his data collection was scheduled right before therapy thus delaying the start of his sessions.

### 3.3. Application of the Behavioral Coding Scheme

#### 3.3.1. Inter-Rater Reliability

Behavioral coding of the home and clinic videos was completed for two participants. Participant 5 was videotaped in the clinic. He had three baseline videos and 11 intervention videos. Participant 7 was the only participant in the second grouping (e.g., #7–#12) who submitted the minimum number of home videos for the application of the coding scheme and the plotting of the data. These videos were used to determine the inter-rater reliability and the feasibility of using the data for a graphic representation of the results.

Inter-rater reliability was strong between the student coders on this project. The percentage agreement calculations were as follows: 90%, 97%, 97%, and 94%, with an overall average of 94%.

#### 3.3.2. Graphic Representation of Coded Data

**Figure 1:**
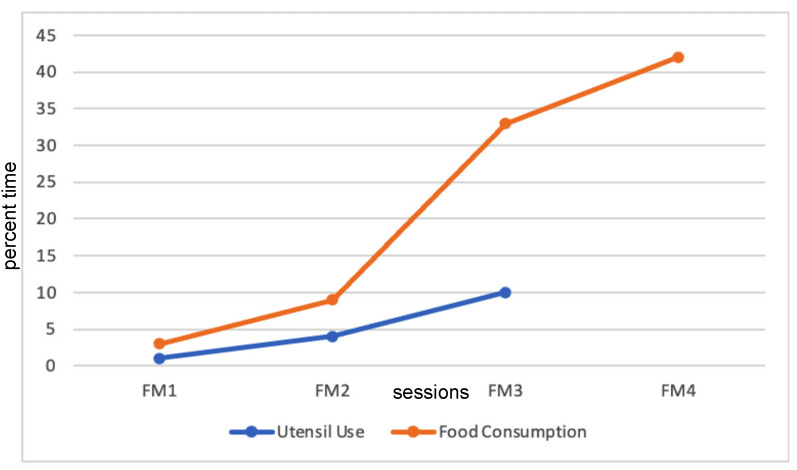
Behavioral Coding Results: Home. Note: FM stands for family meal.

**Figure 2:**
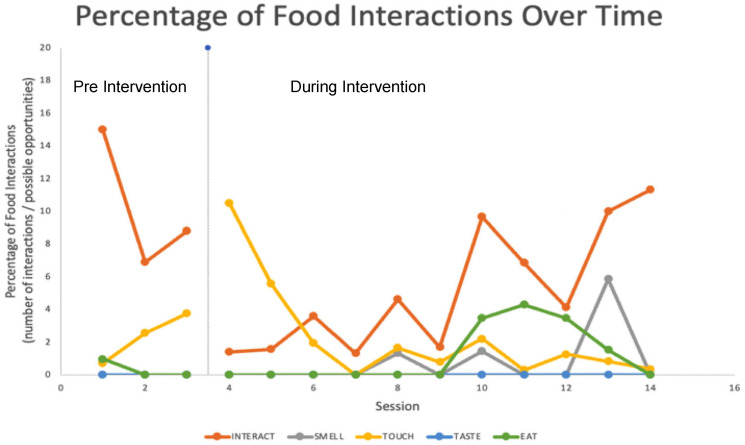
Behavioral Coding Results: Clinic.

Data were represented in a graphic format to demonstrate the feasibility of reporting coded data. Figure 1 and Figure 2 illustrate the potential for the use of the behavioral coding scheme to reflect changes. In Figure 1, for participant 7, the family meal data were reported across the intervention. The data collected show the potential of demonstrating improvements in the frequency of specific parent-generated, operationally defined behaviors. For this child, the home measures included “utensil use for self-feeding” and “food consumption”, which were coded as “eating food during family meals” (FM). Utensil use was operationalized as percent time using a cup, spoon, fork, or knife during mealtime, and food consumption was operationalized as the percentage of mealtime engaged in eating behaviors (e.g., (1) bites food, chews it, and swallows all, or (2) takes puree liquid into mouth and swallows). Utensil use could not be coded for family meal #4, as the foods presented did not require utensils.

Figure 2 demonstrates the feasibility of collecting data during clinic visits. Although data were collected prior to the initiation of the intervention, these data were not reflective of a properly controlled single-subject study. Rather, they provide a graphic representation of these coded behaviors. A single-subject study would require all the baseline data to be stable or in decline before initiating the intervention. The coded data reflected a great deal of variability in the performance across the intervention, with the potential to show changes (i.e., an upward trend in “eats food” and “tolerates smells”).

### 3.4. Pre- and Post-Intervention Measures

The pre- and post-intervention measure data were collected from participant 7 to participant 12 (*n* = 6). A paired sample Wilcoxon signed-rank test was employed to provide preliminary evidence of the sensitivity of the outcomes to reflect change.

#### 3.4.1. Visual Analog Scale

The VAS data are reported in Table 3. The raw data suggest that all questions changed in the expected direction from pre- to post-intervention, with the exception of question #8 “My child’s sensory differences interfere with feeding”. Questions that showed significant improvement included the following: “I can support my child while eating”, “My child can use utensils when eating”, “My child does not get overwhelmed when presented with novel foods”, “My child learns about new foods through exploration”, “My child can eat like other kids his/her age”, “My child has coping strategies to deal with stress when eating”.

#### 3.4.2. Parent Stress Index—Fourth Edition

The subtest scores for the parent and child domain subscales are reported in Table 4 and Table 5. None of the statistical results were significant, although many of the domain subscales changed in the expected direction and were considered useful for further study [33]. Among those close to significant in the parent domain were the subscales in *isolation*, *health*, and *depression*, *followed by competency and role restriction*. The subscales for *attachment* and *spousal/parenting partner relationship* were essentially unchanged. The child domains showed marginal significance in *adaptability* and *demandingness*. Non-significant change in the expected direction was also noted in mood. The subscales that remained relatively unchanged were *distractibility, reinforces parent*, and *acceptability*.

## 4. Discussion

This pilot study addressed three important objectives that will inform future research on the SOS Approach to Feeding. A fidelity measure was created and found to have preliminary inter-rater reliability. Additionally, a behavioral coding scheme was created that included objective behaviors based on the SOS Steps to Eating Food Hierarchy. This hierarchy encompassed 32 steps that are described as components of independent eating and preliminary inter-rater reliability for scoring was achieved. Finally, the sensitive outcome measures were identified; all outcome measures were deemed sensitive to change and thus recommended for future research. Although not a specific aim of the study, videotaping was found to be feasible but presented unique challenges both in the clinic and at home.

A fidelity measure is an essential ingredient of any treatment effectiveness study [34]. To our knowledge, this is the first report on the creation of a reliable fidelity tool for the SOS Approach to Feeding. As highlighted in the literature, treatment fidelity ensures the reliability and internal validity of a study [34]. In designing future studies, treatment fidelity will help to increase scientific confidence in the notion that changes in feeding behavior are due to the initiation of the SOS Approach to Feeding.

The behavioral coding scheme was also a positive next step as the identification of objective outcomes is another important component of treatment effectiveness research. This is particularly true for single-subject case designs that utilize ‘probes’ (i.e., observable behaviors) to graphically represent change. A similar behavioral coding system for measuring feeding outcomes has been reported in the literature [35]. As in this study, behavioral coding is suggested as a valuable outcome measure, as it provides unique insights into behaviors that self-report measures may not address [35]. This study demonstrates that the behavioral coding of the developmental progression of feeding behaviors is both feasible and reliable. Potential exists to use this methodology to show objective improvements and measurable changes at home and in the clinic (i.e., taste, smell, utensil use, and food consumption), indicating that behavioral coding is sensitive to change. Clinic sessions were set up before and during the intervention across the intensive program, and family meal home videos were collected in a naturalistic environment. Benefits were noted in both approaches to collecting data; the clinic can provide a more controlled environment regarding the consistency of procedures employed, while the naturalistic setting for home videos is less controlled and involves less standardization of the meal. The benefits of collecting data in the natural environment are that it provides a view into the participant’s world, provides an objective measure of their progress, and minimizes the risk of the Hawthorne effect [35,36]. Thus, it is recommended that both approaches be included in an effectiveness study.

Difficulty in collecting clinic data was complicated by several factors. First was the challenge of collecting data prior to the initiation of intervention due to the immediate need to start intervention. There was also resistance to the collection of data during the intervention phase. Authors suggest that this resistance was the result of the following two elements: Firstly, sessions were conducted in an evaluative manner and thus were not as fun as therapy. Secondly, the delay in the start of a therapy session seemed to result in a degree of anxiety and annoyance in the child participant. Thus, researchers suggest a different time be targeted in the future for data to be collected, i.e., on a day the child is not receiving therapy.

Home family meal videos were also a concern because of the burden on the family. Using this methodology, families were required to record and submit their own videos, which may have led to increased stress. The home mealtime environment is difficult to control for confounding variables such as environment, foods presented, and consistency in routine [15]. Inconsistencies exist due to the potential presence of preferred versus non-preferred foods and distractions that might exist in the environment. Results can be impacted by factors including the way family members interact with the child, the type of food served, the sensory components of the foods, and the time of day of the meal. Creating specific guidelines instructing the families on how to set up meals and record videos could reduce this variability. The benefits outweigh the increase in variability when collecting data in a natural setting. The changes observed at home may make the results more meaningful to the family, as opposed to the data collected in the clinic in a more controlled clinical setting.

This pilot study demonstrated the sensitivity of the VAS to measure change and confirmed that a VAS may be a useful tool to reflect changes in a way that is not possible within the standardized scales. Results indicated that the questions on the VAS were meaningful to the families in this study. Improvements were noted in their ability to support their child while eating, the ability of the child to use utensils, the tolerance of the child to engage with novel or non-preferred food, the child’s ability to eat in an age-appropriate manner and the child’s coping strategies to cope with stress. Although not statistically significant, the change in the parents’ knowledge of tools and strategies is noteworthy and, along with the change in their ability to support their child, is consistent with the other literature which has shown that the strategies the parents learn in therapy can support the child at home [36]. Surprisingly, one question that did not show change was related to the parents attributing sensory differences to their child’s feeding challenges. This is likely due to the fact that, during intervention, parents learn about other factors [37] (in addition to sensory differences) that are impacting their child’s feeding behavior.

The VAS was found to be a useful method for collecting data as a repeated measure during intervention. Authors suggest that measuring change immediately after intervention may not be reflective of the totality of the gains experienced from the treatment. It is likely that continued improvements can be made once a family returns home, allowing for the consolidation of knowledge and more time for strategies to be integrated into the home environment. Other research within and outside of the current feeding literature supports the measurement of outcomes weeks post-intervention [36,38,39].

Lastly, the PSI also demonstrated the potential to show change. Areas of parental stress that can be considered for further study include *isolation*, *health*, *depression*, *competency*, and *role restriction*. These findings suggest a more positive feeling expressed by the parents about their ability to parent their child, to access social support systems, to engage in a relationship with their child, and to feel more optimistic in performing the role of a parent. Further, the outcomes may indicate that feeding issues and/or the stress that accompanies feeding can impact a parent’s emotions, health, sense of belonging, and ability to respond to the needs of their child. This is consistent with the literature that acknowledges the amount of stress a parent of a child with feeding difficulties experiences [3], as well as the literature that demonstrates a reduction in parental stress from engagement in intervention ([38,40].

The child domain of the PSI appears to capture meaningful changes in *adaptability* and *demandingness*. Further study is warranted, but the results suggest that the parents were better able to adjust to their child’s behavior or that each parent’s stress level was reduced by a change in the child’s behavior (i.e., related to acquiring new feeding habits/routines/skills). Consistent with the previous research, these subscales seem to indicate that raising a child with neurodevelopmental differences can put a strain on one’s parenting skills [20]. Such findings imply that the parents believe that the demands of parenting a child with special needs may surpass their resources to cope [41]. Although the previous studies with the PSI have reported on the parent domain only, this study suggests that the child domain contributes important information about the characteristics of the child that make them difficult to parent, as well as the potential changes in stress that result from gaining knowledge about their child’s strengths and challenges during participation in an intensive feeding intervention program. Similar results were found in a longitudinal study of parental participation in an intervention study for children born prematurely [40]. The parents’ reports of decreased child-related stress appeared to be an important feature of the intervention effects [38,40], although more pilot testing seems to be warranted.

### Strengths and Limiations

This study was designed to pilot the tools and procedures to be used in treatment effectiveness research. Its strengths are reflected in the following procedures that are recommended for future study. A fidelity measure was established for the SOS Approach to Feeding that is available for use in future studies. Behavioral coding appears to be a useful method for repeated measurements. Limitations were noted in the use of videotaping for data collection. Suggested are changes for obtaining home family meal videos, as well as ensuring consistency in the content/foods included in the family meal videos. It is recommended that future studies schedule a telehealth meeting to watch a live family meal, so that there are fewer burdens on the family to record and send in videos. Although the researchers were able to code the videos based on the behaviors observed, limitations exist in the environments and foods selected since differences existed across the meals. Future studies should include basic family meal guidelines for the increased consistency of meal set-up.

Limitations were also noted during the collection of clinic videos. There appeared to be greater stress when feeding treatment sessions followed clinic videotaping. Thus, scheduling should be considered on days when the child does not receive feeding therapy immediately afterwards. Based on the difference in context and its impact on the behaviors observed, this research recommends collecting both clinic and home videos in future studies.

Although both the VAS and PSI reflected important data, a limitation of the study may have been the collection of data immediately following intervention. It is suggested that it may also be beneficial to collect post-test data 1–2 weeks after the family returns home, following the completion of an intensive program. A review of interventions for reducing parenting stress suggests the need to not only document immediate effects but also include the measurement of longer-term gains [42]. Thus, longitudinal data may reflect additional changes and the generalization of skills to the home environment.

## 5. Conclusions

This pilot study was designed to evaluate the components necessary for the implementation of a treatment effectiveness study of the Sequential Oral Sensory (SOS) Approach to Feeding. Data were collected over a 4-year period from children with a range of developmental disorders. A fidelity measure was created to ensure consistency with the principles of the SOS approach to Feeding and preliminary inter-rater reliability. A behavioral coding system, consistent with the SOS Steps to Eating Hierarchy, was developed for scoring objective feeding behaviors, which demonstrated preliminary inter-rater reliability. The visual analog scale (VAS) was sensitive to changes in each parent’s ability to support their child. The Parent Stress Index (PSI) also showed sensitivity to change through a decline in parent-reported stress and child stress indices. Recommended procedures from this pilot study will support future feeding intervention effectiveness research.

## Figures and Tables

**Table 1:** Demographics of participants.

ID	Age	Gender	Diagnosis
001	4	M	Oral Phase Dysphagia; Pediatric Feeding Disorder
002	4	M	Failure to Gain Weight (per Medical History); Oral Phase Dysphagia; Pediatric Feeding Disorder
003	5	M	Undiagnosed Idiopathic Sensory Processing Challenges
004	8	M	ADHD ^a^; Anxiety Disorder; DMDD ^b^
005	7	M	Global Developmental Delay; Tactile and Auditory Over-Responsivity; Vestibular and Proprioceptive Discrimination Challenges
006	5	M	Non-Speaking ASD ^c^; Dyspraxia; Tactile, Auditory, and Visual Over-Responsivity
007	11	M	ASD; ADHD; Generalized Sensory Processing Challenges
008	6	M	ASD; ADHD; Tactile, Auditory, and Visual Over-Responsivity
009	11	M	Anxiety Disorder; Dyslexia; Olfactory, Tactile, and Visual Over-Responsivity; Gustatory and Auditory Discrimination Challenges
010	3	F	Autism; Pediatric Feeding Disorder; Dyspraxia; Vestibular, Proprioceptive, and Gustatory Discrimination Challenges
011	2	F	Feeding Difficulties; Reflux; Generalized Sensory Processing Challenges
012	4	F	Generalized Sensory Processing Challenges; Tactile, Taste, and Smell Over-Responsivity

Note: ^a^ Attention Deficit Hyperactivity Disorder; ^b^ Disruptive Mood Dysregulation Disorder; ^c^ Autism Spectrum Disorder.

**Table 2:** Identification of Probe Behaviors Based on Parent Goals.

ID	Parent Goals	Operational Definitions of Potential Probe Behaviors Based on Mealtime Video
001	- Increase time at table for meals - Increase engagement with food/drink	- Measure number of times child leaves the table, turns face away, or says “no” - Code number # of interactions with food or beverages at table as defined by the Steps to Eating Food Hierarchy
002	- Increase self-feeding skills - Sitting still during mealtimes - Maintaining sufficient caloric intake	- Report number # of times child brings food to mouth using hand or utensil - Report number # of times child uses a re-regulating strategy (i.e., iPad, rocking back and forth, pushing on chair/table) - Parent records caloric intake twice a week
003	- Decreasing the amount of time it takes to eat - Improving success in trying new foods	- Record length of time child takes to each a meal - Code number # instances child tastes, bites or chews a non-preferred foods with or without parent involvement/cueing
004	- Increase range of foods eaten - Increase volume during snacks/meals	- List number of new foods, textures or food types explored at mealtime (includes touching, tasting, smelling, biting, or chewing) - Parent reports caloric intake of snacks daily

**Table 3:** Visual Analog Scale Questions.

Question *n* = 6	Pretest	Posttest			
	M (SD)	M (SD)	*z* Score	*p* Value	Effect Size
Q1: I understand how development impacts feeding	70.17 (20.15)	76.83 (21.67)	0.81	0.42	0.33
Q2: I have an understand of my child's feeding skills, abilities, and capacities	57.17 (21.18)	65.00 (22.73)	0.73	0.46	0.30
Q3: I understand why my child struggles with feeding	71.67 (16.94)	76.50 (16.74)	0.73	0.46	0.30
Q4: I have tools and strategies to support my child in eating successfully	56.67 (22.37)	73.00 (22.37)	1.36	0.17	0.56
Q5: I am able to support my child in eating successfully	49.67 (21.46)	74.33 (21.46)	2.20	0.03	0.90
Q6: My child can use utensils when needed for food	37.50 (26.81)	60.33 (26.81)	2.20	0.03	0.90
Q7: My child does not get overwhelmed when presented with novel or non-preferred foods	35.33 (19.37)	61.67 (19.37)	2.00	0.05	0.82
Q8: I understand how my child's sensory differences interfere with feeding	62.50 (36.24)	56.00 (36.24)	0.73	0.46	0.30
Q9: My child can sit long enough for a meal or snack	56.67 (31.61)	73.33 (31.61)	0.94	0.35	0.38
Q10: My child learns about new foods through exploration or play	51.17 (30.95)	71.33 (30.95)	2.02	0.04	0.82
Q11: My child can eat like other kids his/her age	20.00 (16.31)	46.33 (16.31)	1.99	0.05	0.81
Q12: My child has coping strategies to handle stress when eating	22.80 (18.67)	63.40 (18.67)	2.02	0.04	0.82

**Table 4:** PSI Child Domain Scores Pre- and Post-Intervention.

Child Domain *n* = 6	Pre-Test	Post-Test			
	M (SD)	M (SD)	*z* Score	*p* Value	Effect Size
Distractibility	41.67 (20.72)	39.33 (16.07)	0.73	0.47	0.30
Adaptability	41.17 (11.02)	37.17 (10.91)	1.47	0.14	0.60
Reinforces Parent	23.00 (14.99)	23.33 (14.38)	0.71	0.48	0.29
Demandingness	42.33 (18.07)	35.67 (10.89)	1.79	0.07	0.73
Mood	30.83 (25.38)	28.17 (21.23)	1.15	0.25	0.47
Acceptability	27.67 (13.38)	27.33 (11.64)	0.14	0.89	0.06

**Table 5:** PSI Parent Domain Scores Pre- and Post-Intervention.

Parent Domain *n* = 6	Pre-Test	Post-Test			
	M (SD)	M (SD)	*z* Score	*p* Value	Effect Size
Competency	40.17 (10.94)	35.33 (7.26)	1.58	0.12	0.64
Isolation	30.33 (27.57)	25.00 (22.15)	1.83	0.07	0.75
Attachment	24.83 (15.57)	23.67 (15.96)	0.74	0.46	0.30
Health	25.83 (22.35)	22.33 (18.77)	1.75	0.08	0.71
Role Restriction	34.17 (19.03)	27.50 (18.16)	1.58	0.12	0.64
Depression	36.67 (18.80)	31.17 (15.47)	1.76	0.08	0.72
Spousal Relationship	28.00 (19.91)	26.33 (16.68)	0.41	0.68	0.17

## Data Availability

The original contributions presented in this study are included in the article. Further inquiries can be directed to the corresponding author.
